# Free-Flight Odor Tracking in *Drosophila* Is Consistent with an Optimal Intermittent Scale-Free Search

**DOI:** 10.1371/journal.pone.0000354

**Published:** 2007-04-04

**Authors:** Andy M. Reynolds, Mark A. Frye

**Affiliations:** 1 Rothamsted Research, Harpenden, United Kingdom; 2 University of California Los Angeles, Department of Physiological Science, Los Angeles, California, United States of America; University of Bristol, United Kingdom

## Abstract

During their trajectories in still air, fruit flies (*Drosophila melanogaster*) explore their landscape using a series of straight flight paths punctuated by rapid 90° body-saccades [1]. Some saccades are triggered by visual expansion associated with collision avoidance. Yet many saccades are not triggered by visual cues, but rather appear spontaneously. Our analysis reveals that the control of these visually independent saccades and the flight intervals between them constitute an optimal scale-free active searching strategy. Two characteristics of mathematical optimality that are apparent during free-flight in *Drosophila* are inter-saccade interval lengths distributed according to an inverse square law, which does not vary across landscape scale, and 90° saccade angles, which increase the likelihood that territory will be revisited and thereby reduce the likelihood that near-by targets will be missed. We also show that searching is intermittent, such that active searching phases randomly alternate with relocation phases. Behaviorally, this intermittency is reflected in frequently occurring short, slow speed inter-saccade intervals randomly alternating with rarer, longer, faster inter-saccade intervals. Searching patterns that scale similarly across orders of magnitude of length (i.e., scale-free) have been revealed in animals as diverse as microzooplankton, bumblebees, albatrosses, and spider monkeys, but these do not appear to be optimised with respect to turning angle, whereas *Drosophila* free-flight search does. Also, intermittent searching patterns, such as those reported here for *Drosophila*, have been observed in foragers such as planktivorous fish and ground foraging birds. Our results with freely flying *Drosophila* may constitute the first reported example of searching behaviour that is both scale-free and intermittent.

## Introduction

Shlesinger and Klafter [2] were the first to suggest that the movement patterns of some biological organisms may have so-called “Lévy-flight” characteristics. Lévy-flights, named after the French mathematician Paul Pierre Lévy are comprised of random sequences of movement-segments (such as flying, swimming, or walking), with lengths, l, drawn from a probability distribution function having a power-law tail, *p*(*l*)∼*l^−μ^* where1<*μ*<3. Such a distribution is said to have a “heavy” tail because large-length values are more prevalent than would be present within other random distributions, such as Poisson or Gaussian. A sequence of consecutive lengths drawn from this distribution, connected together and forming a so-called Lévy-flight trajectory, has no characteristic scale because the variance of *p*(*l*) is divergent and therefore is said to be ‘scale-free’. Qualitatively, a Lévy flight is characterized by frequently occurring but relatively short move lengths punctuated by rarely occurring longer lengths, which in turn are punctuated by the rarest longest lengths, and so on. Over much iteration, a Lévy flight will be distributed much farther from its starting position than a Gaussian (i.e. Brownian) random walk of the same length.

The scale-free and super-diffusive properties of Lévy-flights can lead to advantages over Gaussian motions in search scenarios [3]. For example, Viswanathan et al., [4] demonstrated that *μ* = 2 constitutes an optimal Lévy-flight search strategy for locating targets that are distributed randomly and sparsely. Under such conditions, a *μ* = 2 Lévy search strategy minimizes the mean distance traveled and presumably the mean energy expended before encountering a target. The strategy is optimal if the searcher is exclusively engaged in searching, has no prior knowledge of target locations, and if the mean spacing between successive targets greatly exceeds the searcher's perceptual range. A recent mathematical analysis predicts that such optimal conditions exist for searching that is intermittent, which is to say that short active searching phases randomly alternate with long relocation phases [5]. It turns out that Lévy-flights with *μ* = 2 have been found to characterise the movement patterns of a diverse range of animals including, albatrosses, deer, bumblebees [4], [6], jackals [7], microzooplankton [8], spider monkeys [9] and even human hunter-gathers [10]. Also, there is evidence that the walking patterns of some ants, beetles, grasshoppers and spider mites may exhibit a scale-free or fractal property [11]–[13], which is consistent with optimal Lévy flight searching patterns. To date, scale-free characteristics in insect flight patterns have not been reported. Yet, it stands to reason that an animal such as a fruit fly that takes to the wing in search of sparsely distributed food resources such as fallen fruit would benefit from an optimal random Lévy search strategy. The strategy may even incorporate a systematic component such as a persistent sense of turning, as in the case of microzooplankton [8].

During flight in still air, the fruit fly Drosophila melanogaster explores its landscape using a series of straight flight paths punctuated by rapid changes in heading termed “body-saccades” after the functional analogy to the gaze stabilizing eye movements that humans make [14]. During body saccades, fruit flies change heading by 90° over a period of about 50 ms [1]. Many of these free-flight saccades within this experiment are triggered by expanding patterns of optic flow generated upon approach to the high contrast arena wall. Collisions are avoided by saccading away from the center of visual expansion. However, many saccades occur far from the walls of the arena, where visual expansion cues are weak or even invisible through the spatial low-pass characteristics of the fly retina. The properties of these more frequently occurring ‘spontaneous’ saccades have hitherto been unknown. They could be triggered by synaptic and cellular noise within motor circuits or by unknown sensory cues. By contrast, they may reflect a search strategy, patterned within the central nervous system, which evokes changes in flight heading independent of exteroceptive sensory signals. We set out to explicitly test the hypothesis that the spontaneous saccades represent an active, and perhaps mathematically optimized, search strategy. Therefore, we analyzed trajectories for (1) scale-free characteristics, (2) correspondence with μ = 2 Lévy flights, and (3) persistence of turning direction and intermittency of the search and relocation phases. Then we compared trajectories under different visual conditions to determine whether the visual landscape influences the statistics of the search pattern. Finally, we investigate the influence of odor on active search. Our results show that the trajectories constitute a searching strategy that is optimized for locating randomly and sparsely distributed targets (food).

### Collecting free-flight trajectories

All experiments were performed on *Drosophila melanogaster* (Meigen) that had eclosed two or three days earlier from a laboratory stock maintained at Univ. California, Berkeley. Animals were maintained on 12 h∶12 h L∶D photoperiod and tested 5 hours after the onset of subjective day. To motivate long flight sequences, flies were starved for 4 hours and adapted to the light level of the arena for 2 hours prior to each experiment. Upon being released in the arena, each fly generated a continuous flight sequence lasting from several seconds to several minutes before landing on the floor or escaping the arena, at which point data collection was terminated. A 1m diameter, 0.6m high circular arena was illuminated from above with an array of infrared light-emitting diodes. Flight trajectories were monitored by infrared-sensitive cameras and sampled at 30Hz. Each video frame was digitally background-subtracted to enhance the contrast of the fly. Then, the XYZ position of the fly was determined using custom image processing software routines written in MATLAB v.6. For more details on the tracking system, see [1]. For experimental treatments with an attractive odorant, a 0.5 ml tube filled with apple cider vinegar was imbedded in the floor midway between the center and the wall of the arena. The tube was painted black, and mounted flush with the black arena floor to minimize its visibility.

**Figure 1:**
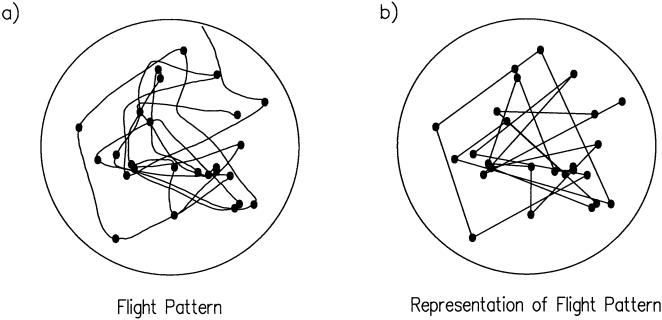
Flight patterns and their representations. a) An example flight pattern of a *Drosophila* within a circular chamber of diameter 1000 mm and depth 600 mm. The trajectory was recorded with a stereo video system at 30 frames per second and lasts for about 11.3 s. There was no odour stimuli and the visual background was uniform and white. b) Representation of a projected flight path as straight-line movements between the positions (•) at which changes in flight direction occurred. A change in flight direction is deemed to have arisen when the direction of the current flight segment (joining two successive recorded positions) and that of the flight segment immediately following the last deemed change in direction, is equal or more than 90°.

The arena was backlit with a circular array of halogen lamps. Mean luminance within the arena was 15 cd/m for all experiments. The arena walls were lined with either a uniform white panorama or printed black and white random array of individual squares each subtending 5° at the center of the arena (50% probability of black). The floor of the arena consisted of black flock paper, which enhanced the contrast of the bright fly on the dark background. The ceiling consisted of a cylinder of dense black cloth, which inhibited an upward phototactic escape response when flies were placed in the arena. The dark floor and ceiling also served to form contrasting horizontal edges with the walls, providing flies with stabilizing cues in all visual landscapes including the uniform white surround. To avoid interference from residual odor stimuli, the visual background patterns and the floor of the arena were replaced after each odor treatment. Once released, individual flies explored the arena using a series of straight flight intervals punctuated by saccades (Fig. 1a). Analyses are based upon the flight trajectories of 11 individuals. Flight durations ranged from approximately 5.3 s to 67.8 s.

## Results

We analyzed the free-flight trajectories for (i) scale-free characteristics, (ii) correspondence with μ = 2 *Lévy* flights, (iii) persistence in turning direction, (iv) intermittency of search and relocation phases, and for (v) the influence of visual and olfactory sensory cues.

### Scale free characteristics

**Figure 2:**
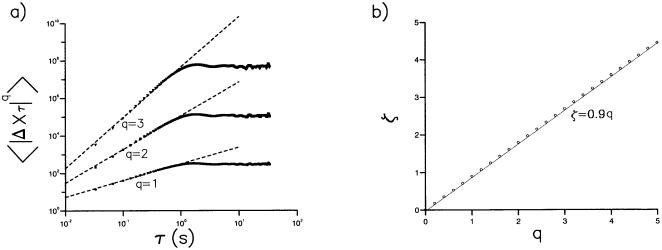
Structure function analysis. a) Structure functions 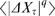 characterizing the displacements, Δ*X_τ_*, in a time increment *τ* of hungry *Drosophila* in an odorless arena with white walls (symbols). *Drosophila* flights were projected onto the horizontal (x-y) plane. Power-scaling of the structure functions is indicated (dashed line). This scaling was obtained from least squares fitting of logarithms of the structure functions to logarithms of the time intervals, *τ*, for*τ*≤1*s*. b) Scaling-exponents, *ζ*, obtained in this manner are typically prescribed with a standard error of about ±0.01*q* and Pearson's correlation coefficient, *R* ^2^ = 0.99 Scaling exponents (symbols, o) are well represented by *ζ* = 0.9*q* (solid line) (*R* ^2^ = 1).

Our first test for the presence of a scale-free characteristics involves the displacements 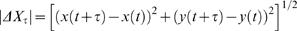 made by the flies within time intervals, *τ*. Here *x*(*t*) and *y*(*t*) are the coordinates, in the horizontal plane, of an individual fly at time, *t*. We calculated the moments, 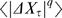, of these displacements by ensemble averaging over all 11 trajectories. These moments have a functional dependency upon the time interval and are called ‘structure functions’. A power-law relationship of the form 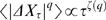 where *ζ = αq* would be indicative of scale-free behaviour [15]. Structure functions for *Drosophila* flight displacements exhibit power-law scaling behaviour with *α*≈0.9 when the time increment *τ* is less than about 1 second, or when the time increment *τ*, corresponding to the video sampling interval 33 milliseconds, is less than the average time to fly cross the arena in our experiments (minimally 3-seconds) (Fig. 2). The turnover in scaling above *τ* = 1*s* is therefore spurious in the sense that it does not reflect the scaling of unconstrained, freely roaming *Drosophila* which can have inter-saccade intervals lasting for more than1*s* [16], [17].

**Figure 3:**
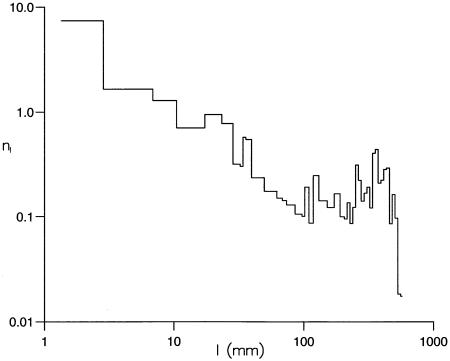
The distribution, *n_l_*, of lengths, *l*, of ‘straight-line flight segments’ (inter-saccade intervals). The sizes of the data collection bins are logarithmically distributed and numbers of straight-line flight segments have been normalised by the bin sizes. The straight line with slope -1.3 constitutes a linear least squares fit to the data for 10<*l*<100 *mm* (Pearson's correlation coefficient, *R* ^2^ = 0.87).

To facilitate the analysis we followed Bartumeus *et al*. [8] and represented the trajectories as sequences of straight-line segments between body saccades. (Fig. 1b). The distribution of straight-line segment lengths, representing inter-saccade intervals, does not immediately reveal inverse-square power-law(*μ* = 2) scaling indicative of scale-free behaviour (Fig. 3). Instead, there is the resemblance of power-law scaling regime with *μ* = 1.3 and the presence of a secondary maximum that arises from flight segments which span the entire experimental chamber and are truncated at the walls of the experimental chamber. The truncation of many shorter length segments that arises close to the walls because of collision avoidance may be preventing the detection of inverse-square power-law scaling in this test. The results of subsequent examinations presented below confirm that this is indeed the case.

**Figure 4:**
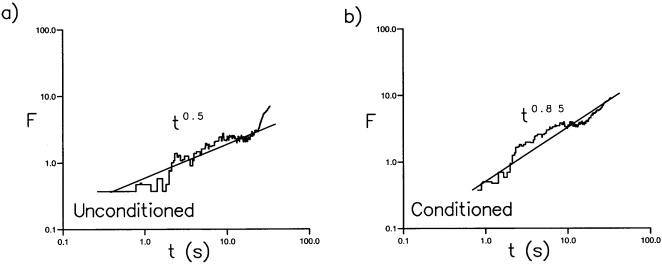
The net root mean square displacement, F is plotted as a function of elapsed time *t*, measured from the first recorded positions of the *Drosophila*. a) The unconditional analysis is based on all turns within the 3-dimensional flight patterns. The straight line with *β* = 0.5 constitutes a linear least squares fit to the data (Pearson's correlation coefficient, *R* ^2^ = 0.90). b) In the conditional analysis a turn is deemed to have arisen only if it is located at least 150 mm from the walls of the chamber. Many turns are that associated with collision avoidance are thereby excluded in the conditional analysis. The straight line with *β* = 0.85 constitutes a linear least squares fit to the data (*R* ^2^ = 0.90). The value of the scaling exponent, *α*, does not depend sensitively upon the distance of 350 mm and statistically indistinguishable values are obtained for distances in the range 300 to 400 mm.

Our next examination uses the fact that the number of turning points occurring within time intervals *t* to *t*+Δ*t* define a dimensionless time series, *u*(*t*), and an associated dimensionless ‘displacement’ 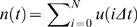. If the values of *n*(*t*) are completely uncorrelated and behave like ‘white noise’, then the root-mean-square displacement 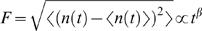 where *β* = 1/2 and where the angular brackets denote an ensemble average over all 11 trajectories in the data set [18]. Short-term correlations in the data may cause the initial slope of a plot of log *F*/log *t* to differ from ½ (i.e.*β*≠½), although it will still approach ½ over the long-term. However, long-range correlations with no characteristic scale [18] will generate *β* values≠½. For our flight data, *β*≈0.85once turns occurring less than 150 *mm* from the walls, and therefore likely associated with collision avoidance manoeuvres, are discounted (Fig. 4). The same estimate for the value of *β*is obtained when the critical distance, 150 *mm*, is varied by ±50 *mm* (data not shown). These results suggest that the flight behaviour near the center of the arena and therefore not associated with collision-avoidance maneuvers, is not represented by a characteristic scale.

**Figure 5:**
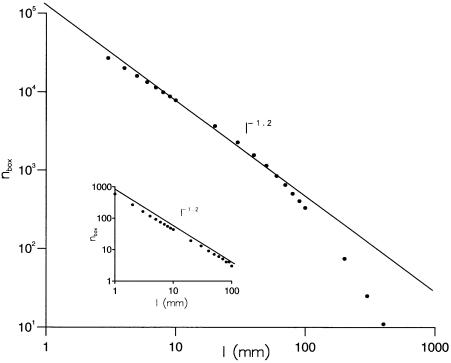
Assessment of the fractal dimension associated with the representations of the *Drosophila* flights. The average number, *n_l_*, of boxes of size *l_box_* required to enclose the representations of the 3-dimensional *Drosophila* flights is plotted against *l_box_* (dots). A power-law relationship of the form 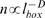 would be indicative of a scale-free characteristic with fractal dimension D, and here, a linear least squares fit shows that D = 1.2. Fractal scaling is evident for spatial scales between about 5 and 75 mm. The insert shows the same plot for a series of simulated Lévy-flights with *μ* = 2.

We complete our scale analysis with an assessment of the fractal dimension associated with the representations of the *Drosophila* trajectories. The average number,*n_l_*, of boxes of size *l_box_* required to enclose the flight trajectories plotted against *l_box_* (Fig. 5) contains a power-law relationship of the form 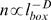, which is indicative of a scale-free characteristic of fractal dimension D. Fractal scaling with *D* = 1.2 is evident for spatial scales between about 5 and 75 mm (Fig. 5). To briefly summarize, we have demonstrated that the *Drosophila* trajectories are scale-free by showing that (1) displacements have a power-law dependency upon time of flight, (2) the time series of saccade intervals has a long-range power-law correlation, and (3) trajectories are fractal.

### Correspondence with *μ*≈2 Lévy Flights

Flight trajectories comprising a series of straight flight paths punctuated by rapid body-saccades, together with the presence of occasional long inter-saccade flight paths, suggests that Lévy-flights underlie the searching strategy of freely flying *Drosophila*. Our results are consistent with this proposition. Lévy flights produce structure functions with power-law scaling that is characterized by *ζ = αq* where *α* = 1/(*μ*−1) [15]. *Drosophila* trajectories displayed such scaling with *α*≈0.9 (Fig. 2) and are therefore consistent with the presence of Lévy flights with *μ*≈2.1. The scaling exponent*β* characterising the time series of turns made by Lévy flights approaches the limit 2−*μ*/2 asymptotically for sufficiently long sequences [6] whilst the fractal dimension of *D* approaches *μ*−1. However, finitely long Lévy flights with *μ* = 2.0 correspond to *β*≈0.85 [6] and *D* = 1.2 (Fig 5, insert). These values of *β* and *D* characterise the saccade time series data (Fig. 4) and the fractal dimension of the *Drosophila* trajectories (Fig. 5). Hence the values of *α*, *β* and *D* derived from the results of separate analyses are consistent with the presence of *μ*≈2 Lévy-flights. This is consistent with previous works that have shown power-law distributions of activity in the fly's “motor cortex” [19], [20].

### Persistent turning direction

**Figure 6:**
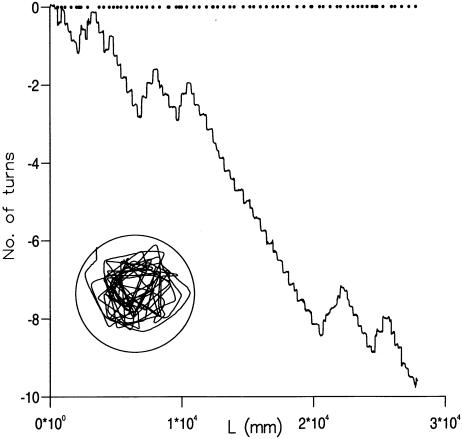
An example of the cumulative number of ‘saccade cycles’ made by a *Drosophila* as a function of the distance, L, flown. One ‘cycle’ corresponds to a series of body saccades in which the animal's heading changes ±360° within the arena. Positive cycles are made in clockwise direction whilst negative cycles are made in an anticlockwise direction. The flight pattern consists of straight-line flight-segments punctuated by saccades (Insert). The locations of the saccades along the flight path are indicated(•).

All of the recorded *Drosophila* flight trajectories exhibited consistent turning direction, either clockwise or counterclockwise, over at least 1000 mm, corresponding to 4 or more consecutive saccades (e.g. Fig. 6). These consecutive uni-directional trajectories crudely resemble spirals. However, the sides of these apparent spirals have random lengths and therefore differ from systematic spiral searching patterns which have, for example, been observed in the desert isopod *Hemilepistus reaumuri* upon getting lost after an excursion from its burrow [21] and in desert ants (*Cataglyphis*) when returning to the nest after forging beyond the range of the known landmark map [22]. These latter systematic spiral trajectories increase the tendency for an animal to encounter new territory, and thereby avoid “missing” the home. By contrast, the persistent sense of turning exhibited by *Drosophila* has the advantage of reducing the probability of missing nearby targets by increasing the likelihood that territory will be revisited [8].

**Figure 7:**
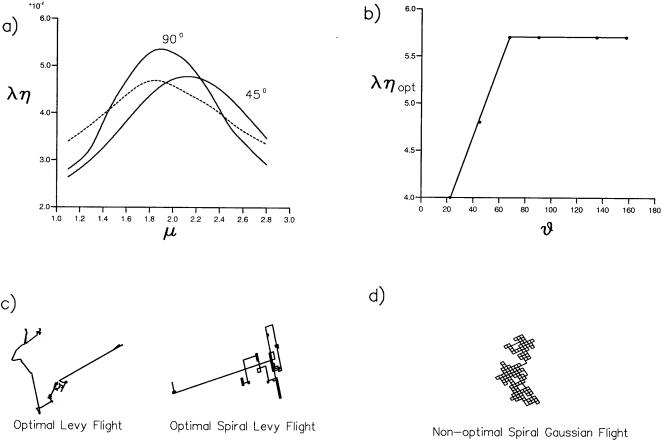
Searching efficiencies and persistent turning. a) Searching efficiencies, *η*, for the location of targets placed at = the vertices of a square lattice with spacing, *λ*, as a function of the Lévy exponent, *μ*, characterising the distribution of flight segment lengths. The searching efficiency is the reciprocal of the mean distance travelled before first encountering a target. Simulation data are shown *λ*/*r* = 100 where r is the range at which a target can be detected for the case when flight-segments are randomly orientated (dashed-line) and for the case when the angle between successive flight segments is 45° and 90° (solid lines). Searching commences from just beyond (r, r). The searching is optimal when *μ*≈2 and when the turning angle is equal to or greater than 90°. The results of simulations (not shown) reveal that this is also optimal for *λ*/*r* = 10 and *λ*/*r* = 1000. Spiralling Lévy flights remain optimal if the sense of turning switches back and forth between 90° and −90° after completing two or more turns of the spiral. More frequent switching leads to a searching strategy that is less efficient than Lévy flights with randomly distributed turning angles. b) Simulation data for the optimal searching, efficiency, *η_opt_* = max(*η*(*μ*)), as a function of the turning angle, *ϑ* (•). The solid-line is added to guide the eye. c) *μ* = 2 Lévy flights with random turns and with 90° turns. The spiralling promotes the revisiting of territory and thereby reduces the probability that nearby targets will be missed. d) A non-optimal Gaussian (*μ* = 4) spiral flight pattern.

For this strategy to be effective in flies, the animals must not only exhibit consistent saccade *direction* in order to spiral, but also must exhibit consistent saccade *amplitude*. Mean saccade angle in *Drosophila* is 90 degrees [1]. Our own numerical simulations (Fig 7a) for the searching efficiencies of fruit flies show that saccade angle should be constant at −90 and 90 degrees. This is in stark contrast with the original model in which predicted angles between successive flight segments are randomly and uniformly distributed between 0° and 360° [4]. The previous report considered an idealised model in which a searcher moves on a straight line towards the nearest target if the target site lies within a ‘direct vision’ radius, *r*, otherwise the searcher chooses a direction at random, and then a travel distance, *l*, is drawn from a Lévy distribution. The simulation moves incrementally towards the new location whilst seeking targets within detection radius, *r*. If no target is sighted, the searcher stops after traversing the distance *l* and chooses a new direction and a new distance, otherwise it proceeds to the target [4]. Here we modified the model so that the angle between successive straight line segments is constant. Searching efficiencies were determined from the simulated trajectories of 10^5^ searchers that began their searches in the immediate vicinity of a target. When the target is low (*L*/*r*≥100) the mean distance travelled, and so the energy expenditure, is a minimum when *μ*≈2 and when the turning angle equal to or larger than 90° (Fig. 7). This new result complements that of Viswanathan *et al.* [4] and may account for the 90° saccade amplitudes exhibited by *Drosophila* [1], [23].

### Intermittency

**Figure 8:**
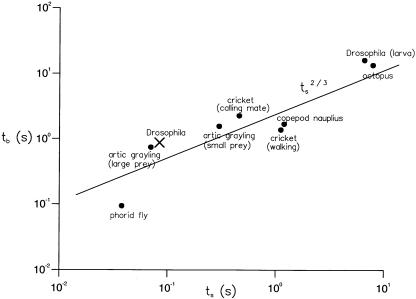
Mean times in the local active searching phase, *t_s_*, comprised of short flight-segments having length *L*<30 *mm* and in the relocation phase, *t_r_* (X), comprised of long flight-segment having length *L*>30 *mm*. Mean times do not change significantly when the length scale *L* is increased or decreased by a factor of 2. Mean times for a diverse range of organisms with intermittent locomotion (•) (phorid fly, general locomotion; arctic grayling, food search with small and large prey; cricket, spontaneous walking and in presence of a calling mate; copepod nauplius, general swimming with food present; *Drosophila meglanogaster* larva, crawling on a non-feeding substrate; octopus, moving over reef while foraging) [30]. The scaling relation *t_r_*∝*t_s_* ^2/3^ predicted by the Lévy-flight model of optimal searching [5] is shown (solid line).

To overcome their own inertia, fruit flies actively decelerate prior to executing saccades, and accelerate afterwards [24]. Therefore, very short saccade intervals are generally associated with low flight speeds and large intervals with high speeds [24, Fig. 6]. However, flies often decelerate *more* prior to short saccade intervals than prior to long ones. For all saccade intervals less than *30 mm*, the mean flight speed during the first 1/30 s of flight is *285 mm/s* (*s.d. = 193mm/*s) while for intervals longer than *30 mm* it is *356 mm/s* (*s.d. = 183mm/s*) (Student's t-test, *p<0.05*, degrees of freedom *n = 239, t = 2.47*). This suggests that the slow-down is not only related to inertial constraints, [24] but rather may also reflect a controlled search strategy in which flies decelerate more in order to maximize sensory feedback during the following short flight interval. The average times that *Drosophila* maintain slow, short ‘searching’ versus fast, long ‘relocation phases’, as compared with the same measurements for a diverse range of creatures, obey the scaling relation, *τ_r_*∝*τ_s_* ^2/3^ (Fig. 8) and are therefore consistent with the scale-free model of intermittent searching [5].

### Influence of visual and olfactory sensory cues

We recorded the flight behaviour of 16 flies within an arena lined with a high-contrast random checkerboard panorama. We recorded flies within the arena lined with a uniform white panorama, at the same luminance. Flight durations ranged from 9.9 to 194.0 sec. Under these two visual conditions, the general characteristics of flight do not vary, except that owing to stronger collision-avoidance visual cues flight trajectories tend to be distributed closer to the center of the arena for the high-contrast visual treatment [1]. The scaling exponents, *α* = 0.9 (Pearson's correlation coefficient,*R* ^2^ = 0.99) and *β* = 0.85(*R* ^2^ = 0.90) and fractal dimension, *D* = 1.3, that characterized trajectories recorded within the low-contrast arena were exactly the same as those, *α* = 0.9, *β* = 0.85 and *D* = 1.3, characterizing flight trajectories within the high-contrast arena. Therefore, varying the contrast and therefore the magnitude of optic flow stimuli in the free-flight appears to have no influence on Lévy search properties.

**Figure 9:**
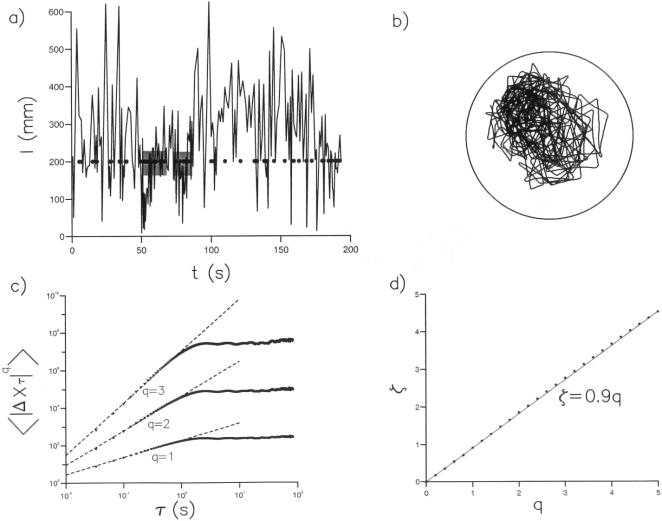
Flight patterns in chamber with an odor source. a) An example of a sequence of intra-saccade flight lengths, *l*, for a *Drosophila* within an arena containing an odor source and lined with a high-contrast random checkerboard panorama (solid-line). Saccades occurring within 100 mm of the odor source are indicated (•). Sustained bouts of short length flights tend to occur in the vicinity of the odor source. The most pronounced of these occur between 50 and 60 s, and between 70 and 80 s (within shaded boxes). These bouts interrupt the Lévy flight searching. b) A projection of the flight pattern. The odor source is hidden in the floor of the arena and is located in the top left-hand quadrant of the projection. c) Structure functions 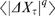 characterizing the displacements, Δ*X_τ_*, in a time increment *τ* of the *Drosophila* flights projected onto the horizontal (x-y) plane. d) Power-scaling of the structure functions is indicated (dashed line). This scaling was obtained from least squares fitting of the structure functions for *τ*≤1*s*. Scaling-exponents, *ζ*, obtained in this manner are typically prescribed with a standard error of about ±0.01*q* and Pearson's correlation coefficient, *R* ^2^ = 0.99 Scaling exponents (symbols, •) are well represented by *ζ* = 0.9*q* (solid line) (*R* ^2^ = 1).

By contrast, the presence of an attractive odor source had a profound influence over Lévy flights. Introducing an odor source hidden in the floor of the arena resulted in a robust and repeatable change in the trajectories when the visual background was textured, a prerequisite for odor localization [23]. Under this visual condition, most flies eventually located the odor source and subsequently spent more of their time flying back and forth over it (Fig. 9a,b). The freely roaming Lévy searching strategy that is evident when flies are far from the odour source (Fig. 9 c,d) and characterized by *α* = 0.9, *β* = 0.85 and *D* = 1.3, disappeared when flies flew into the vicinity of the source, where they instead executed slow, short flight inter-saccade flight segments more reminiscent of Gaussian (Brownian) length distributions (Fig 9a,b). Other quantitative shifts in flight behaviour accompanying successful odour location, including a decrease in flight altitude and more frequent approaches to visually textured walls of the arena near to the odour source, are described in detail in Frye *et al*., [23].

## Discussion

When searching for food, fruit flies (*Drosophila melanogaster*) explore their landscape using a series of straight-line flight paths punctuated by rapid 90° turns called saccades (Fig. 1). Some of these saccades are associated with collision avoidance. The purpose of the more frequently occurring ‘spontaneous’ saccades has hitherto been unknown but could form a systematic searching strategy to enable flies to localize the source of attractive odors emanating from fallen fruit. Thus we tested whether free-flight trajectories represent an active, optimized, search strategy. Individual flies were released in a 1m diameter arena under varying visual and olfactory conditions, and their trajectories were recorded in three dimensions. Saccades were detected programmatically, and we examined the distribution of inter-saccade distances flown over a range of time intervals. The results revealed that the trajectories are ‘scale-free’ (Figs. 2, 4, 5). This means that the distance flown between successive saccades does not have a typical value. Instead, the ‘average’ inter-saccade distance depends upon the duration of the flight record because it is determined by the most rarely occurring and therefore longest of the inter-saccade distances. A ‘random walk’ analysis (Fig. 4) and a fractal analysis (Fig. 5) indicate that the trajectories of fruit flies are well represented by scale-free Lévy-flights. These are comprised of independent straight flight segments, the lengths of which are distributed according to an inverse-square law.

The Lévy exponents or fractal dimensions of flight trajectories were not dependent upon whether or not the background was visually textured. Nor were they dependent on the presence of an odor stimulus until the animals came very close to its source. When the background was visually textured, most flies located the source of odor and subsequently spent more time flying back and forth over it (Fig 9b). Once the animals approach the odor, the freely roaming Lévy search strategy is abandoned in favour of a more localized (Brownian) flight pattern. The loss of fractality during flight mirrors that seen in the walking patterns of *Drosophila* upon encountering a food odor [25]. A similar shift in searching behaviour occurs in larger flies. Murdie and Hassell [26] showed that the “tightness” of walking houseflies (*Musca domestica.*) searching for sugar droplets increases within the vicinity of the sugar source. However, these walking analyses did not reveal Lévy characteristics.

The disclosure of the power-law function of flight segment length is significant because such movement patterns are known to constitute a mathematically optimal search strategy for “targets” (i.e. odor sources) that are randomly and sparsely distributed [4]. When implemented behaviourally, such a strategy minimizes the mean distance travelled before detecting a target. Not only segment length, but also turn (saccade) angle can be optimized. Several studies (e.g. [4]) have proposed an idealised model of searching in which the angles between successive flight-segments are randomly and uniformly distributed between 0° and 360°. By contrast, we show here for the first time that searching efficiencies are enhanced markedly if the fruit flies make either 90° or −90° saccades over extended portions of their trajectories (Figs 1, 6, 7). By promoting the iterative revisiting of territory, constant saccade angles reduce the likelihood that near-by targets go undetected.

Finally, inter-saccade flight speed is generally lower prior to the execution of shorter length inter-saccade flight segments. It stands to reason that this slow-down cannot be attributed to a failure to recover flight speed reduced during a saccade because it *precedes* the following flight segment. Rather, there is likely to be some active control over flying slowly when turning frequently. This finding suggests that searching is intermittent in character with slow, short, active searching phases randomly alternating with fast, long, relocation flights. Similar intermittent “search-relocate” behaviors have been observed in *Drosophila* walking patterns [25], [27], [28], where the authors suggest that the walking patterns are fractal (i.e. are scale-free). It would appear that fruit flies adopt a scale-free intermittent searching strategy both during walking and flying, and that the strategy is independent of the visual structure of the environment. Since it appears that robust visual feedback is necessary for successful odor localization [23], but there is no influence of visual background on the Lévy flight patterns, then perhaps the influence of visual feedback on odor localization is restricted to the non- Lévy regimes adopted very close to the odor source. This important hypothesis remains to be explored but may shed valuable light on how cross-modal cues are used during foraging.

### Intermittent, Lévy-flight and efficient animal foraging

Either scale-free searching movement patterns or intermittent searching have been identified in a variety of animals [4], [6], [7], [8], [29], [30]. However, our results with freely flying *Drosophila* may be the first reported example of a searching behaviour that is both scale-free and intermittent. This suggests, in accordance with recent theoretical developments [5], that scale-free and intermittent behaviours are not manifestations of two distinctly different kinds of searching strategy, but rather are constituent parts of a single, complex, widely adopted searching strategy (Fig. 8). This result is particularly exciting because it holds the promise of a unified theory for the movement patterns of foraging animals.

### Neural and behavioural mechanisms for generating Lévy-flights

Our results suggest that *Drosophila* generate Lévy flights during free-flight. The next challenge is to determine how this mathematical strategy is implemented by the nervous system. What are the cellular and cell-system mechanisms? Where in the brain does the control circuit reside? What are the neurophysiological underpinnings? Segev *et al*., [31] recently reported that Lévy-stable distributions with an inverse-square law tail characterise the electrical activity of some neuronal networks. Such spontaneous electrical activity could provide the timing signals necessary for the execution of Lévy-flights. In fruit flies, locomotor activity is coordinated by a region called the central complex, casually referred to as the insect's “motor cortex”. Martin *et al.* [19] found that blocking synapses within the ellipsoid-body, a sub-region of the central complex, leads to a loss of the fractal (i.e. Lévy-like) properties of adult walking behavior. They concluded that fractal patterns of locomotor activity are somehow regulated in the ellipsoid-body. It would therefore be fruitful to investigate whether genetically inactivating the ellipsoid body also interrupts the Lévy structure of flight behaviour.
